# Impact of COVID-19 on utilization of maternal, newborn and child health services in Nigeria: protocol for a country-level mixed-methods study

**DOI:** 10.12688/f1000research.26283.2

**Published:** 2021-08-18

**Authors:** Godwin Akaba, Osasuyi Dirisu, Kehinde Okunade, Eseoghene Adams, Jane Ohioghame, Obioma Obikeze, Emmanuel Izuka, Maryam Sulieman, Michael Edeh

**Affiliations:** 1 College of Health Sciences, Department of Obstetrics and Gynaecology, University of Abuja, Abuja, Nigeria; 2 Department of Research, Population Council, Abuja, Nigeria; 3 Department of Obstetrics and Gynaecology, College of Medicine, University of Lagos, Lagos, Nigeria; 4 Research Hub Africa, Abuja, Nigeria; 5 Research and Statistics, Lifesworth- Research Lab II, Abuja, Nigeria; 6 Department of Community Medicine/Public Health, Federal Medical Centre, Yenagoa, Bayelsa, Nigeria; 7 Department of Obstetrics and Gynaecology, University of Nigeria, Abuja, Nigeria; 8 Department of Obstetrics and Gynaecology, Muhammad Abdullahi Wase Teaching Hospital, Kano, Nigeria; 9 Department of Obstetrics and Gynaecology, General Hospital, Takum, Taraaba, Nigeria

**Keywords:** Impact, COVID-19, Utilization, MNCH, Healthcare, Services, Nigeria, Africa

## Abstract

**Background: **Battling with COVID-19 and providing essential services along the continuum of care could be challenging. This study will evaluate the impact of COVID-19 on utilization of maternal, newborn and child health (MNCH) services in Nigeria and explore the barriers being experienced by women and their families in getting access to MNCH services, as well as other contextual factors that may have shaped the utilization of MNCH services during the COVID-19 pandemic.

**Methods and analysis:** The study will adopt an observational mixed-methods study design involving 18 health care facilities delivering MNCH services in six selected states across six geopolitical zones of Nigeria. We will retrieve longitudinal data on MNCH services from all selected hospitals six months before and after the first recorded case of COVID-19 in Nigeria. Qualitative data will be collected using in-depth interviews conducted via mobile phones or ZOOM meeting platforms among stakeholder participants (users of MNCH services, health workers and policymakers) to ascertain their perceptions on how COVID-19 has shaped the utilization of MNCH services. We will triangulate quantitative and qualitative data to better understand the impact of COVID-19 on the utilization of MNCH services in Nigeria.

**Ethics and dissemination:** Ethics approvals have been obtained from the Health Research Ethics Committee of the tertiary hospitals involved in the study. Our findings will provide the first evidence from an African setting on the impact of COVID-19 on the utilization of MNCH services using a mixed-methods study design for policy formulation towards sustained MNCH service delivery.

## Introduction

The Sustainable Development Goals agenda is part of the global efforts to improve maternal, new-born and child health (MNCH) by challenging countries to make efforts to reduce the global burden of maternal, new-born and child mortality ^1^. However, despite these efforts, global maternal mortality remains unacceptably high with about 303,000 women dying each year from pregnancy-related complications ^2^, for which sub-Saharan Africa and Southern Asia accounts for about 86% of such deaths ^1^. According to the World Health Organization (WHO) report, an estimated 5.3 million under-5 deaths and 2.5 million neonatal deaths were reported in 2020, which are equivalent to an under-5 mortality rate of 39 per 1,000 live births and neonatal mortality rate of 18 per 1,000 live births, while the maternal mortality rate is 211 per 100,000 live births ^3^.

In Nigeria, MNCH outcomes have improved over the last decade. However, these outcomes are still rated as being among the worst in the world ^4^. The under-5 mortality rate is 132 deaths per 1,000 live births while the infant mortality rate is 67 deaths per 1,000 live births ^4^. On the other hand, the maternal mortality ratio in Nigeria is 512 per 100,000 live births and some of the main causes of maternal deaths include obstetric haemorrhage, infection, obstructed labour, unsafe abortion, preeclampsia/eclampsia, malaria, anaemia, and contributory factors such as lack of awareness about complications in pregnancy, inability to seek timely medical intervention, lack of utilization of health care services, lack of transportation and inability to pay for services ^4, 5^. With these indices, Nigeria is the second largest contributor to maternal mortality worldwide accounting for more than 10% of all cases ^2, 5^.

In order to improve child survival and maternal health, the continuum of care for MNCH was designed and this has gained so much attention because it is expected to reduce the burden of maternal deaths, neonatal deaths and deaths among under-5 children particularly in lower and middle-income countries ^6, 7^. The continuum of care includes healthcare services for mothers and children from pre-pregnancy to delivery, postnatal and childhood ^6, 8^. Furthermore, this depicts the link between maternal and child health, particularly as they seem to be affected by the same variables. Therefore, this makes it important that at every specific point in the continuum of care, every mother/child pair should receive the full package to attain maximum satisfaction ^6^. Towards a positive impact on overall MNCH indicators, every woman is expected to continuously receive antenatal care (ANC), skilled birth attendance at delivery and postnatal care (PNC). The WHO recommends that every pregnant woman is expected to have a minimum of eight ANC contacts before delivery ^9^. This is because ANC helps to identify pregnancy risks, provide appropriate care for women who might be at risk of potentially fatal conditions, provide opportunities for counselling these women and give access to health-promoting services (weight measurement, blood pressure measurement etc), and it also helps to increase subsequent use of maternal and child health services ^7, 10^. Even though the majority of maternal and neonatal deaths occur in the postpartum period, in Nigeria, only about 42% of mothers utilize PNC services within the first two days after delivery. Similar trends are seen in the utilization of ANC, the number of deliveries attended by skilled birth attendants and uptake of immunization and family planning services. According to the Nigerian Demographic and Health Survey 2018, only 57% of women had at least four ANC visits, deliveries by skilled birth attendants were 43%, immunization coverage for age-appropriate vaccinations was 21%, and modern contraceptive prevalence rate among currently married women was 12% ^4^.

Low utilization of MNCH services can be categorized into two major factors, which are the user factor and the health system factor. The user factor includes distance to a health facility, cultural beliefs and practices, economic factors and many other factors within the community and respective households. On the other hand, the health system factor includes the capacity of health staff, attitude of care providers, infrastructure and other forms of interactions that take place within the healthcare system ^1^.

With the recent COVID-19 pandemic, the resilience of health systems’ and countries’ emergency preparedness and response have been tested. According to the WHO, over 200 countries, areas and territories have been affected and as such health systems have been critically affected ^11^. As of May 17, 2020, there have been 4,534,731 cases of COVID-19 and over 307,537 deaths globally. In Africa, about 58,663 cases and 1,710 deaths have been recorded. On the other hand, in Nigeria, 5,621 cases and 176 deaths have been recorded ^12^. COVID-19 pandemic and its devastating impact on the health systems, livelihood and economies of Nations have been likened to a “war” situation. Health care for women and children are usually disproportionally and negatively impacted at times of war and conflicts. Battling with COVID-19 and providing essential services along the continuum of care is critical and challenging ^4^. To avert deaths, it is important to ensure that appropriate care is available to every woman and every child during the pandemic. We, therefore, hypothesized that COVID-19 pandemic in Nigeria may have further worsened the already poor maternal and child health services utilization in Nigeria. Thus, this study is designed to evaluate the impact of COVID-19 on the utilization of MNCH services in Nigeria using a mixed-method approach. The quantitative part of the study will involve the collection of longitudinal data on maternal and newborn health services, while the qualitative component will explore the barriers being experienced by women and their families in getting access to MNCH services as well as other contextual factors that may have shaped the utilization of MNCH services during the COVID-19 pandemic. It would also explore the level of preparedness of the Nigeria health system in responding to such pandemics and examine the available policies and action plans that are currently in place. Findings from this study will be beneficial in providing key stakeholders at all levels of the health care system with the necessary information that will provide new direction and policy for the country towards mitigating the negative impact of COVID-19 on the already fragile health system, and thus help in sustaining previous efforts towards attaining the third sustainable development goal of “achieving good health and well-being for all” in Nigeria.

## Aim and objectives

The study aims to assess the rate of utilization of maternal and newborn child health (MNCH) services before and during the COVID 19 pandemic as well as to critically examine the various barriers and facilitators associated with accessing MNCH services during the COVID-19 pandemic in Nigeria. Specific objectives are to:

1. Ascertain the trend in the utilization of MNCH services before and during COVID-19 pandemic.
2. Determine the barriers and facilitating factors associated with access to MNCH services during the COVID-19 pandemic.
3. Identify gaps in the MNCH continuum of care and the most affected level of care during the COVID-19 pandemic.
4. Explore contextual factors that may have shaped the utilization of MNCH services during the COVID-19 pandemic

## Protocol

### Study settings

This study will be conducted in six states across the six geopolitical zones of the country. The states were purposively selected during the proposal development and categorized according to the number of reported COVID-19 cases as “high-burden states (high number of cases) and low-burden states (low number of cases)”. For this study, any state with at least 56 confirmed cases of COVID-19 as at May 17, 2020 (equivalent to approximately 1% of total cases of COVID-19 at that time) will be regarded as a high-burden state, while those with less than 56 confirmed cases will be classified as low-burden states. The high-burden states include Lagos in the South-west (n=2,373), Kano in the North-west (n=825) and Abuja FCT in the North-central (n=397), while the low-burden states are Enugu in the South-east (n=11), Taraba in the North-east (n=17) and Bayelsa in the South-south (n=6). The participating states and health facilities are shown in Table 1.

**Table 1.** List of participating states and health facilities.

Level of health facility	High COVID-19 burden states			Low COVID-19 burden states		
	*Abuja FCT*	*Lagos*	*Kano*	*Taraba*	*Enugu*	*Bayelsa*
Tertiary health centre	University of Abuja Teaching Hospital, Gwagwalada, Abuja	Lagos University Teaching Hospital, Idi-Araba, Lagos	Muhammad Abdullahi Wase Teaching Hospital, Kano	Federal Medical Center Jalingo, Taraba	University of Nigeria Teaching Hospital, Ituku- Ozalla, Enugu	Federal Medical Center, Yenagoa
Secondary health centre	General Hospital, Kwali	General Hospital, Badagry	Wudil General Hospital, Wudil	General Hospital, Sunkani Ardokola	Poly General Hospital, Enugu, North LGA	Diete-Koki Memorial Hospital, Opolo
Primary health centre	Comprehensive Health Center, Abaji	Comprehensive Health Center, Isolo	Comprehensive Health Center, Kiru	Rifkatu Danjuma maternity PHC, Takum	Comprehensive Health Center Obukpa, Nsukka	Amarata Primary Health Care Center

### Study design

The study will use a mixed-methods design to evaluate the impact of the novel COVID-19 pandemic on the utilization of MNCH across six selected states of Nigeria. The quantitative part of the study will involve the collection of longitudinal data from hospitals in the participating States on maternal and newborn health services (antenatal clinic attendance, hospital deliveries, postnatal clinic attendance, family planning services and immunization uptakes) six months before (September 1, 2019, to February 28, 2020) and after (March 1 to August 31, 2020) the first recorded case of COVID-19 in Nigeria. The data from the three high-burden states (Abuja FCT, Lagos, Kano) will be compared with that of the three low-burden states (Enugu, Taraba and Bayelsa). The qualitative arm will assess the perceptions of users of healthcare facilities, health workers, and policymakers on how COVID-19 has shaped the utilization of MNCH services in their locality as well as other contextual factors contributing to the projected views.

### Participant recruitment and eligibility criteria

Participants for the qualitative survey will be identified, screened for eligibility, and selected by purposive sampling method by the study coordinator in each of the selected states. The participants will include policymakers, service providers and service users from each of the participating states. Research collaborators in the six states who are all healthcare practitioners working in the tertiary health facilities will support the recruitment of policymakers and service providers in the states. The service providers will help in the recruitment of the service users who presented for MNCH services during the study period.

Inclusion and exclusion criteria for stakeholder enrolment are the same across all participating states. Eligible participants are those aged at least 18 years with adequate physical, mental, and cognitive capacity who give informed consent to participate in the study.

*Definition of stakeholders*

- Policymaker – a healthcare professional who has been working within the health system for at least one year and is involved in defining policy on MNCH services.
- Service provider – a healthcare professional who has been working within the health system for at least one year and involved in the direct provision of MNCH services in any of the study facility before and during the current COVID-19 pandemic.
- Service user – someone who has accessed MNCH services before or during the COVID-19 pandemic.

Qualitative data will be collected by in-depth interviews (IDIs) from the three stakeholder groups comprising of one policymaker, health care worker and service user related to MNCH services from each of the three levels of the healthcare system in the six selected states. The interviews will be conducted using mobile phones or ZOOM application platforms depending on the choice of the respondents due to the social distancing regulations in Nigeria. Nationally, a total of fifty-four (54) IDIs will be conducted (comprising of 18 IDIs for each of the stakeholder groups).

Before the IDIs, the interviewers will be required to check if the participants meet the inclusion criteria. Stakeholders who failed to meet the inclusion criteria or those who declined consent will be excluded from participating in the study. The list of stakeholders to be interviewed and their distribution across the six participating states is as shown in Table 2.

**Table 2.** Stakeholders to be interviewed.

Stakeholder	FCT	Lagos	Kano	Taraba	Enugu	Bayelsa	Total
**Policy maker**	3	3	3	3	3	3	18
**Health care worker**	3	3	3	3	3	3	18
**MNCH service user**	3	3	3	3	3	3	18
**Total**	9	9	9	9	9	9	54

### Theoretical frameworks

**Figure 1.**
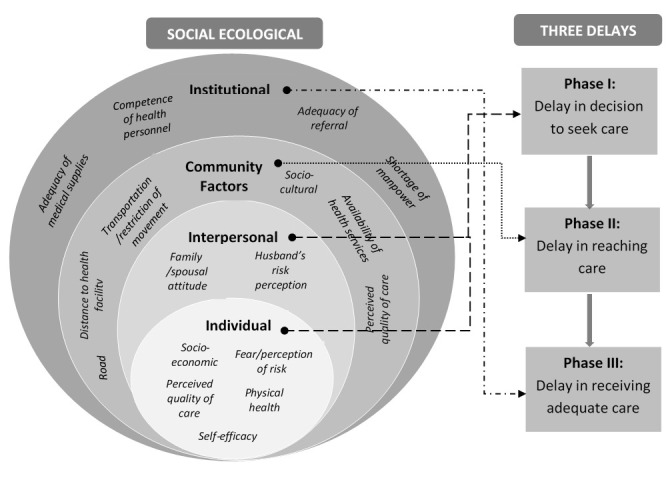
Summary of interactions between the social ecological model, three delays model and utilization of MNCH services.

 Several theoretical frameworks have been developed that may have relevance to exploring and understanding barriers to the utilization of health care services. We will use a hybrid of the social-ecological model (SEM) and the three delays model to investigate and explore how COVID-19 has impacted on utilization of MNCH services in Nigeria. The SEM provides a theory-based approach to understanding the complex interaction between individual, interpersonal, community, institutional and policy factors that influence behaviour and practice ^13^. The delay model recognizes three phases (delay in deciding to seek care, delay in reaching healthcare facility and delay in receiving adequate care at the health facility) as contributing to women’s ability to receive care towards averting poor outcomes. These delays are shaped by several complex factors operating at the same or different levels of delays ^14– 16^. Figure 1 shows the theoretical frameworks to be used for this study.

### Methods of data collection

*Quantitative data*: Data for the three months preceding the first confirmed case of COVID-19 in Nigeria (December 1, 2019, to February 28, 2020) and the three months after (March 1 to May 31, 2020) will be collected from one facility each at the three different levels of the Nigerian healthcare system (primary, secondary and tertiary facilities) in each of the six selected states. The facilities are also to be spread across three different Local Government Areas in each of the participating states. Monthly data to be collected includes: total antenatal care (ANC) attendance, total facility deliveries, total postnatal clinic (PNC) attendance, total family planning services rendered, and the total number of babies who received immunization. These data will be used to evaluate the trends in the utilization of MNCH services in these states.

*Qualitative data*: The state study coordinators will schedule and confirm the dates and time of the planned IDIs with the study participants after obtaining informed written consent. The participants will also be informed that the interview will be recorded during the informed consent process. Interviews will then be facilitated by a trained interviewer over the phone or via ZOOM. The interviewers have expertise in the conduct of IDIs and will be working with members of the core research team to schedule interviews with the respondents while determining the best time for the interview to take place. The study participants will be informed about the purpose of the study and are invited to participate in the interview, which will last for approximately 20 to 30 minutes. The interviews will be conducted using an IDI guide designed specifically for each of the stakeholder groups. After the interview, the audio recordings would be transcribed verbatim. The transcripts will be compared with the audio recordings to ensure completeness and are then analysed and utilized to develop the study report. Table 3 shows examples of some of the question that will be asked for each of the stakeholder groups.

**Table 3.** Examples of Interview guide questions for the different stakeholder groups. MNCH, maternal and newborn child health.

Policy maker	Provider of MNCH services	User of MNCH services
Kindly explain what you know about COVID-19 pandemic?	Kindly explain what you know about COVID-19 pandemic?	Kindly explain what you know about COVID-19 pandemic?
What measures have been put in place as regards MNCH services since the pandemic started?	What are the MNCH services readily available in this facility in this period of COVID-19 pandemic	Have you had any health education session for COVID-19? If yes, kindly explain in details and state how helpful it was.
Are there key policies, strategies and action plans for MNCH services since the pandemic started? (kindly state them) If yes, what is the implementation status? If no, what are the plans to develop one?	Kindly list the MNCH services that is being accessed by clients this period. (State reasons)	What are the MNCH services that are readily available in this facility in this period of COVID-19 pandemic
	Were there MNCH outreach programmes carried out this period?	Kindly list the MNCH services that you accessed this period. (State reasons)
	Were there alternative strategies for immunization activities or other MNCH outreaches?	Have you observed any change as regards accessing MNCH services this period? If yes, what are they? (State reasons for such observed changes)
What are the quality assurance checklists or tools for ensuring quality delivery of MNCH services during this pandemic?	Has there been any observed change as regards provision of MNCH services this period? If yes, what are they? (State reasons for such observed changes)	How safe do you think it is for women to access MNCH services in this facility this period?
What the challenges/barriers that are being experienced in delivery of MNCH services this period?	How safe is it for women to access MNCH services in this facility this period?	Do you think this facility is well equipped to provide MNCH services during this pandemic? (Give reasons)
How can these challenges/barriers be mitigated?	What is the available standard protocol for attending to women who access MNCH services in the ongoing pandemic?	What are the challenges/barriers you experienced accessing MNCH services during this period?
	Do you think this facility is well equipped to provide MNCH services during this pandemic? (Give reasons)	
	Were there referrals this period?	
	Kindly explain some of the coping/adaptive mechanisms that has helped you while providing services.	
	What are the challenges/barriers this facility has experienced in providing MNCH services during this period? (How can they be mitigated)	

### Data management

*Quantitative data:* This data will be entered into a computer and analysed using SPSS version 20.0 for Windows (Armonk, NY: IBM Corp.) to show the trends of the utilization of MNCH services over the six months review period. The differences in MNCH services utilization between the study period (before and during the COVID era) and COVID-19 infection burden (high- and low-burden states) within and across the different levels of the healthcare system will be tested using the independent sample t-test. The level of significance will be reported at *P*<0.05.

*Qualitative data:* Audio recorded interviews will be transcribed verbatim and transcripts will be anonymized with pseudonyms. Data analysis will begin with the reading of transcripts repeatedly to achieve immersion and derive codes by identifying words in the text that capture key concepts. Initial codes will be discussed by the study team and sorted into categories based on linkages between key concepts. The themes that represent key issues with MNCH services will be developed for initial coding and inductive analysis. The data will then be entered into NVivo software version 12 for organization and analysis. Each transcript will be coded by two members of the research team independently to reduce inter-coder variability.

*Data protection:* On completion of this study, transcripts will be stored in password-protected computers/laptops and only the core research team will have access to the data. All members of the team will sign a confidentiality agreement. All study participants will be assigned an identification code, which will be delinked from their identity at the data entry point. Audio recordings will be destroyed at the end of the project. Transcripts will be stored for a minimum of 5 years after the project ends and will only be destroyed afterwards if necessary.

## Ethical considerations

Ethics approvals have been obtained from the Health Research Ethics Committee of the participating tertiary hospitals involved in the study: University of Abuja Teaching Hospital (UATH/HREC/PR/2020/001), Lagos University Teaching Hospital (LUTHHREC/EREV/0520/42), University of Nigeria Teaching Hospital, Enugu (NHREC/05/01/2008B-FWA00002458-1RB00002323), Mohammad Abdullahi Wase Teaching Hospital, Kano (MOH/Off/797/T.I/2060), Federal Medical Centre, Yenagoa, Bayelsa (FMC/ADM/017/Vol.1/138), Federal Medical Centre, Jalingo, Taraba (FMC/JL/ADM/330). Social approval and permission will be sought from the primary and secondary facilities selected for the study. The ethical review undertaken by all project investigators will ensure standard processes (dignity, autonomy, informed consent, confidentiality, anonymity, ability to adhere to protocol) and data security are maintained. Voluntary and informed participation, confidentiality and safety of participants will constitute key principles of researcher–respondent interaction. Written consent will be obtained from service users, service providers and policymakers prior to their enrolment in the study. The study will be conducted according to the Helsinki declarations on ethical principles for medical research involving human subjects.

## Patient and public involvement, and dissemination

The development of the research question was based on experiences of patients narrated in the media, as well as during medical consultations at the obstetric emergency services of the University of Abuja Teaching Hospital, Abuja. Their inputs were sought in designing the qualitative component of the study proposal.

Findings from this study will be disseminated by delivering presentations at national and international conferences, organization of study dissemination meetings involving relevant stakeholders in Nigeria and publishing articles in peer-reviewed journals. Additionally, all participating institutions and patients will be notified of the key findings from the study. Data resulting from this study will be deposited in a data repository (Mendeley Data) and can be assessed by the public.

## Study status

The study is currently collecting both longitudinal data and qualitative data.

## Discussion

This paper reports a protocol for an observational mixed-methods study design aimed at collecting quantitative data showing the trend of utilization of MNCH services during the three months preceding and during the COVID-19 pandemic, and qualitative data from key stakeholders across the health systems in the six geopolitical zones in Nigeria to determine the barriers and facilitators associated with access to MNCH services during the COVID-19 pandemic. Additionally, the study will explore other contextual issues that have shaped service delivery along the MNCH continuum of care during the COVID-19 pandemic. The study findings will provide a timely contribution to the ongoing debate on the magnitude of impact on access and utilization of MNCH services across the different levels of the Nigeria health system following the COVID-19 outbreak and also help in identifying strategies that will assist Nigeria and other African countries in maintaining focus towards the realization of the third sustainable development goal of “ensuring good health and well-being for all” amidst the COVID-19 pandemic.

## Conclusion

The identification of the state of MNCH continuum of care and the most affected components of care during the COVID-19 pandemic in Nigeria could lead to multiple benefits for service users, service providers, policymakers and other relevant stakeholders towards effective planning for the strengthening of national health systems during the COVID-19 pandemic.

## Data availability statement

No data are associated with this article.
