# Supplementary material for: Impact of COVID-19 on utilization of maternal, newborn and child health services in Nigeria: protocol for a country-level mixed-methods study
Source: F1000Res. 2021 Aug 18;9:1106. Originally published 2020 Sep 9. [Version 2] doi: 10.12688/f1000research.26283.2 (PMC8422339; doi:10.12688/f1000research.26283.2)
Supplement: Peer review history

# Peer review: Reviewer response for version 2

**DOI:** 10.5256/f1000research.76594.r92146

**Published:** 2021-09-06

**Authors:** Marleen Temmerman

**Affiliations:** 1 Centre of Excellence in Women and Child Health East Africa, Aga Khan University, Nairobi, Kenya

I have reviewed the paper and can agree with the comments/changes and the authors responses.

Is the study design appropriate for the research question?

Partly

Is the rationale for, and objectives of, the study clearly described?

Yes

Are sufficient details of the methods provided to allow replication by others?

Yes

Are the datasets clearly presented in a useable and accessible format?

Yes

Reviewer Expertise:

RMNCAH, clinical, epidemiological, health systems, policy

I confirm that I have read this submission and believe that I have an appropriate level of expertise to confirm that it is of an acceptable scientific standard.

# Peer review: Reviewer response for version 1

**DOI:** 10.5256/f1000research.29010.r75059

**Published:** 2021-01-11

**Authors:** Marleen Temmerman

**Affiliations:** 1 Centre of Excellence in Women and Child Health East Africa, Aga Khan University, Nairobi, Kenya

Well designed protocol to address an important public health issue that is the utilization of essential health services, more specifically mother and child health services.

 The proposed methodology adopts quantitative and qualitative mixed-methods approach, addressing users of services, health care providers and policymakers.

**Comments:**

- The proposed timeframe of 3 months before and e months after the first covid case seems short and probably does not allow the current status following the initial 'covid-19 shock'.
- The voices missing are the women that do not attend the MCH services anymore; they should be included in FGDs.

Is the study design appropriate for the research question?

Partly

Is the rationale for, and objectives of, the study clearly described?

Yes

Are sufficient details of the methods provided to allow replication by others?

Yes

Are the datasets clearly presented in a useable and accessible format?

Yes

Reviewer Expertise:

RMNCAH, clinical, epidemiological, health systems, policy

I confirm that I have read this submission and believe that I have an appropriate level of expertise to confirm that it is of an acceptable scientific standard, however I have significant reservations, as outlined above.

# Response

**Published:** 2021-01-12

**Authors:** GODWIN AKABA

**Affiliations:** Dr., Nigeria

Dear Reviewer,

 Thank you for your comments. I wish to respond that our study received ethical approvals in the first and second weeks of June 2020 at which time Nigeria was only about 3 months into the COVID -19 pandemic and we commenced data collection on 22nd June 2020 to 30th September 2020. The objective of our study was to investigate the effect of lockdown due to the COVID-19 pandemic on the utilization of MNCH services in Nigeria. This protocol was first submitted to this Journal (F1000 Research) on 20th  August 2020 at which time the authors had declared that we were already collecting both longitudinal and qualitative data which describes fully the situation of COVID-19 in Nigeria at that time during which Nigeria was still in lockdown. We believe that if this research protocol was reviewed at that time the comment may not have been made since the data were collected within 4- 6 months of the COVID-19 pandemic in Nigeria.

 Thank you

 Godwin.O.Akaba

# Peer review: Reviewer response for version 1

**DOI:** 10.5256/f1000research.29010.r71048

**Published:** 2020-10-05

**Authors:** Ngozi Orazulike, Adedayo Tella

**Affiliations:** 1 School of Public Health, University of Port Harcourt, Port Harcourt, Nigeria; 2 Department of Obstetrics and Gynaecology, University of Port Harcourt Teaching Hospital, Port Harcourt, Nigeria; 3 Centre for Medical Research and Training, College of Health Sciences, University of Port Harcourt, Port Harcourt, Nigeria

Thank you for the invitation to review this article at its proposal stage.

**Summary:**

 This article seeks to evaluate the impact on utilization of maternal, newborn and child health (MNCH) services in Nigeria by the COVID-19 pandemic as battling with COVID-19 and providing essential health care services can be a real challenge. The study will adopt a mixed-methods study design involving 18 health care facilities delivering MNCH services at the 3 levels of care in six selected states across the six geopolitical zones of Nigeria over a six month period (pre and post first recorded case of COVID-19 in Nigeria). The findings from this study will aid policy formulation towards sustained MNCH services during the COVID-19 pandemic and beyond.

**Comments to the authors:**

 An interesting and well thought-out study.

 Three months before and during COVID-19 pandemic may not give the entire picture of MNCH services but to avoid recall bias, the duration covered is appropriate. In terms of use of the term ‘rates’ the duration is not up to a year, as such projections may be considered. We have few suggestions;

 Grammar:

1. Many conjunctions are used erroneously. ‘However’ and ‘on the other hand’ should be used for divergent views and not for views with similar flow of information.
2. Some sentences are too long Paragraph 3, lines 9 -13; also rephrase Paragraph 2; lines 3-7.
3. Paragraph 3, line 3; low income countries not lower.
4. Use of past and future tenses need to be checked. A continuous check throughout the write up is required. This is a protocol but some events have taken place and some will take place in the future.

Introduction:

1. Maternal, newborn and child health (MNCH) - What of the period of infancy? Infancy should be included because of the peculiarity of this disease that affects infants and their caregivers (usually mothers) during that period.
2. Paragraph 3, line 13; The sentence, 'Even though the majority of maternal and neonatal deaths occur in the postpartum period in Nigeria, only about 42% of mothers....' is contentious. Is majority of maternal death really postpartum or peri-partum? Give a reference for the 42%.
3. Rate of utilization – rate is usually with an annual denominator (time), yours is over 6 months or are you projecting?

Protocol:

1. Under Study design, specify the type of study. This is a cross sectional study using a mixed-methods approach.
2. Under Study design, can the authors specify how the longitudinal data will be collected from hospitals in the participating states. Will the data be sampled or will data of all patients that access MNCH services in those states be collated? Is it in retrospect? We believe this should be addressed to make the article scientifically sound.
3. Under Study design, line 8; '....Contextual factors...' – Give some explanation or examples.
4. What approach will you be using for the qualitative study? – phenomenological?
5. Under Methods of data collection (qualitative), check this sentence; 'The interview will be recorded during informed consent---' wording can be simply put as audio recording of informed consent.
6. In-depth interview for 20 minutes is regarded as short, about 30 minutes; or 30-40 minutes is preferable.
7. Was the IDI guide tested and if yes, where and among which stakeholders?
8. Under Patient and public involvement and dissemination; dissemination to participants and other stakeholders should come first before disseminating to national and international conferences, because the activity serves as both a dissemination and verification exercise when it is among participants of a study.
9. Under Data management (Qualitative); Do you intend to code before entering the transcript into NVivo or import the transcript and code in NVivo? 
10. Under Data Management (Quantitative); Use of t-test for proportions? Consider Chi-square test for proportion.
11. Under Data Protection; Audiotapes destroyed immediately after the project could be risky. Keeping in a safe place for 6 months before destruction may be safer.

Thank you.

Is the study design appropriate for the research question?

Yes

Is the rationale for, and objectives of, the study clearly described?

Yes

Are sufficient details of the methods provided to allow replication by others?

Partly

Are the datasets clearly presented in a useable and accessible format?

Not applicable

Reviewer Expertise:

Fetomaternal medicine, infertility and assisted conception, reproductive health.

We confirm that we have read this submission and believe that we have an appropriate level of expertise to confirm that it is of an acceptable scientific standard.
